# The Sir Titus Salt Hospital

**Published:** 1894-02-24

**Authors:** 


### THE SIR TITUS SALT HOSPITAL.

The famous little township in the valley of the Aire, known as Saltaire, named after and built by that famous philanthropic British workman, manufacturer, millionaire, and baronet, the late Sir Titus Salt, has among its many excellent institutions a hospital, though small, equal in comfort and management to any in the three kingdoms. At the corner of one of the avenues in the model city is a small building, in the prevailing architecture of the place. On presenting my card at the door I was ushered into a bright little hall and taken into the Matron's room—a cheery, cosy sitting-room. A kettle was singing on the hob, and the nursing staff, the Matron and Sister, were taking tea. It was a cheerless afternoon without. The two ladies, in their smart nursing garb, pouring out the steaming Ceylon, made a veritable black and white picture, which seemed to thaw the icy chill the dreary winter weather had cast over me. The little hospital was in keeping with the cosy room of the Matron-nurse. On the ground floor were the waiting-room, surgery and operating-room, and the dispensary. There were only nine beds in the wards above. The hospital originally started with six beds when it was opened in 1868, but since then one of the almshouses next door has been absorbed to the advantage of the hospital. Sir Titus Salt was very much to the front with his fine old presentment on the walls in each room; and over a child's cot was a portrait of another gentleman, to whom in many ways generous, good old Sir Titus had some resemblance, that of Santa Claus.

